# Use of a biological mesh for the treatment of perineal fistula following radical colorectal resection

**DOI:** 10.1002/ccr3.1346

**Published:** 2018-03-30

**Authors:** Marinos C. Makris, Michael Kornaropoulos, Apostolos Krikelis, Demetrios Moris, Diamantis I. Tsilimigras, Elia Modestou, Artemis Liapi, Vasileios Karatzias, Christos Damaskos, Andreas Zevlas

**Affiliations:** ^1^ 1^st^ Surgical Department General Hospital of Athens “G. Gennimatas” Athens Greece; ^2^ Alpha Institute of Biomedical Sciences (AIBS) Marousi Athens Greece; ^3^ Department of Surgery The Ohio State University Comprehensive Cancer Center The Ohio State University Columbus Ohio; ^4^ School of Medicine National and Kapodistrian University of Athens Athens Greece; ^5^ N.S. Christeas Laboratory of Experimental Surgery and Surgical Research Medical School National and Kapodistrian University of Athens Athens Greece

**Keywords:** Biological mesh, fistula, multiple colorectal operations

## Abstract

Patients with postradiation therapy for malignancies and/or extensive colorectal surgery are prone to the development of enteroperineal fistulas. Application of biological meshes may prove beneficial in treating complicated enteroperineal fistulas as they provide a stable ground for closing pelvic defects even in contaminated fields.

## Introduction

The surgical management of enterocutaneous fistula (ECF) has been challenging; few data on their treatment have been published to date and no official guidelines exist 1, 2. There is also absence of commonly accepted protocols regarding the nutritional management of these entities 3.

Enterocutaneous fistulas are abnormal communications usually between the small or large bowel, and less frequently between the stomach and the skin of the abdominal wall or the perineum. Patients with postradiation therapy for malignancies and/or extensive rectal surgery are prone to the development of such fistulas 2. Among the difficulties of the operative treatment following the debridement of the pelvic cavity during abdominoperineal resection (APR) are the resulting abdominal wall defects or dead space on the perineal floor.

The utilization of meshes during the last decades has offered a viable solution to the surgeons regarding the repair of defects on such “hostile” surgical fields 1. Synthetic meshes have been inadequate due to the formation of adhesions with the viscera and the high possibility of fistula recurrence; however, accumulating evidence has shown that biocompatible grafts such as biological meshes can be applied successfully 4. Herein, we present a patient with a complex perineal fistula following multiple operations at the pelvic area for colorectal cancer and anastomotic recurrence.

## Case History

A 43‐year‐old male patient was admitted to our clinic with a diagnosis of adenοcarcinoma of the lower sigmoid colon. The computed tomography (CT) of the abdomen scan had not demonstrated metastatic foci. His past medical history was unremarkable. The patient underwent low anterior resection (LAR). On the 7th postoperative day, he presented with acute abdominal pain with rebound tenderness and fecal content coming out from the drainage. The CT scan showed an accumulation near the anastomosis. Ηe underwent an urgent laparotomy due to fecal peritonitis and leakage from the anastomosis. A temporary loop transverse colostomy and a reconstruction of the sigmoid‐rectal anastomosis were performed. The postoperative period was uneventful, and the patient was discharged in optimal condition. After oncologic consultation, the patient received combination of radiotherapy and chemotherapy.

Three years later, the patient was readmitted to the hospital because of rectal bleeding, dull abdominal pain, and intermittent lumbar pain. A biopsy performed during colonoscopy revealed recurrence of the tumor on the anastomosis close to the dentate line. The patient underwent abdominoperineal resection after extensive adhesiolysis, and a terminal colostomy of the descending colon was created. On the 7th postoperative day, the patient underwent urgent laparotomy due to fever and rebound tenderness and an avulsion of the jejunum was identified, which was repaired by two layers of suture. The remaining intestine was intact. He had an uncomplicated postoperative course.

**Figure 1:**
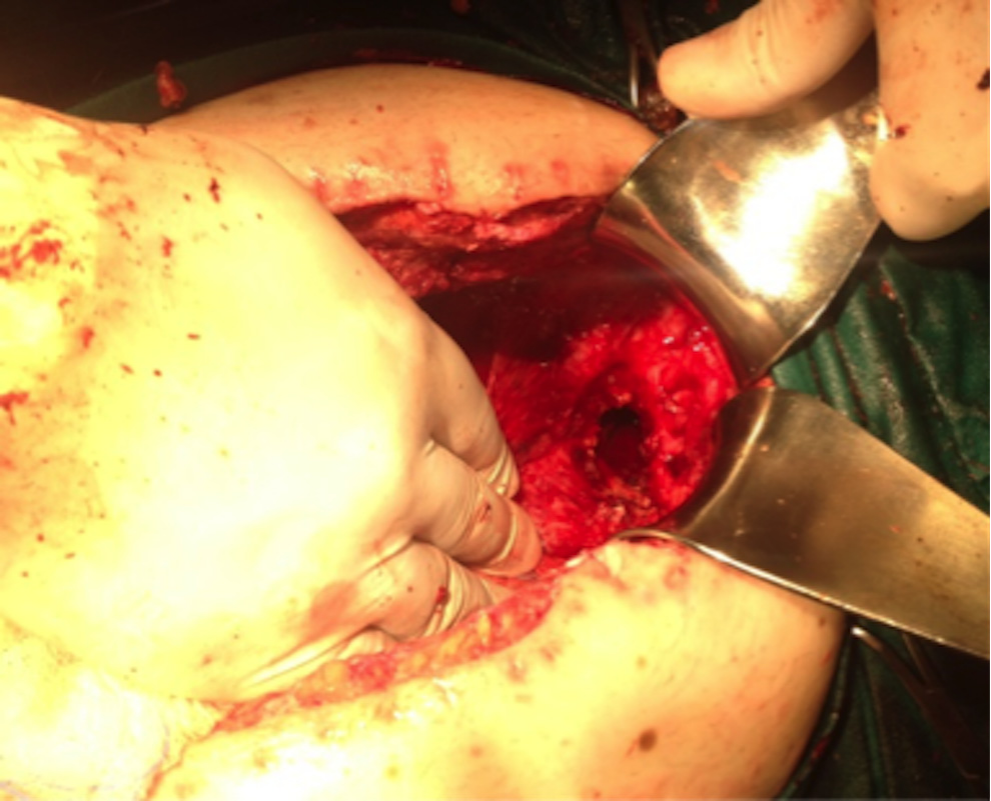
The pelvic outlet leading to the empty perineal space.

One month later, the patient presented to the hospital with symptoms of urinary tract infection and an ECF of low to moderate output volume (200–500 mL/day) with a visible orifice on the perineal skin. He was treated conservatively with total parenteral nutrition (TPN) and antibiotics; the ECF's output gradually decreased and finally disappeared. The patient was discharged and readmitted with recurrence of the ECF 5 months later. This time, the external orifice was a defect, which was 3 cm in diameter, on the gluteal cleft. The output was high (>500 mL/day) causing electrolyte disturbances and local skin maceration. The patient was operated again; after extensive adhesiolysis, small bowel lacerations occurred and multiple enteroenteric fistulas were detected. These vulnerable segments were partially resected. During the procedure, one of the small bowel loops was identified, protruding to the perineal space and seemed to create the internal orifice of the fistula. The loop was reduced to the peritoneal cavity, and a segmental resection with an ileo‐ileal anastomosis was performed near the ileocecal valve creating a defunctioning loop ileostomy. During the procedure, an empty space anteriorly to the presacral fascia in the perineal space with size almost 3 × 3 cm was revealed (Fig. 1). A continuous absorbable suture 2‐0 Vicryl on the pelvic floor was applied to avoid the protrusion of the intestine in this “dead” space. The short postoperative course was uncomplicated and the patient was discharged on the 10th postoperative day.

**Figure 2:**
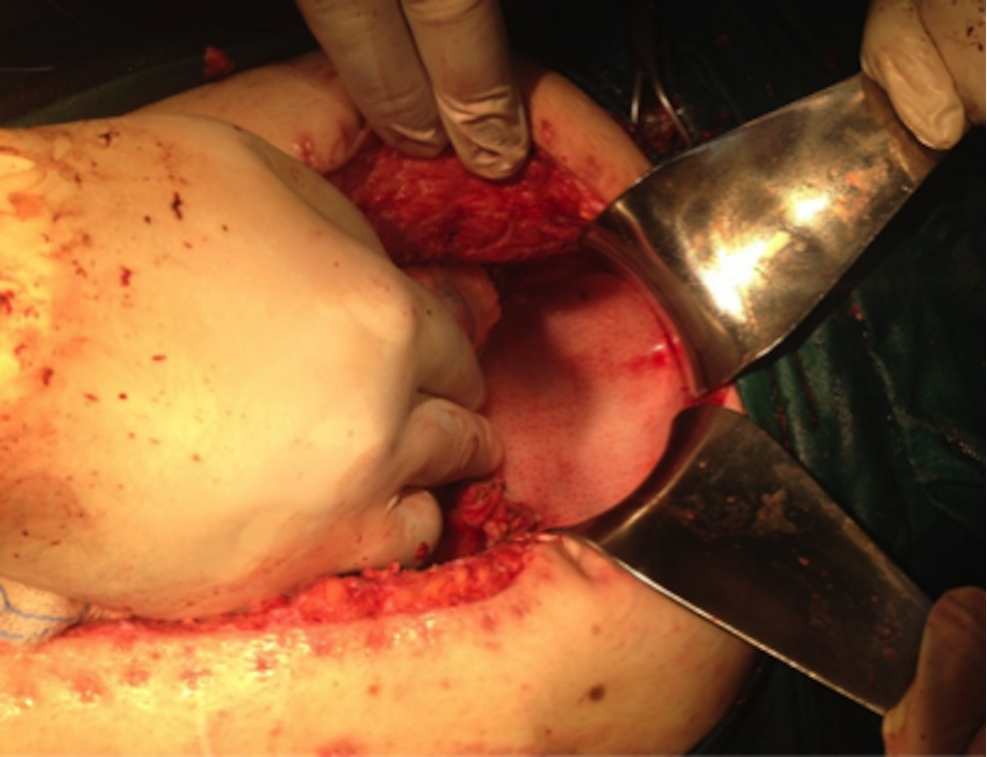
The pelvic outlet “sealed” after application of a biological mesh.

One month later, the patient presented with recurrence of the high volume output fistula and underwent an urgent laparotomy once again. After extensive adhesiolysis, loops of terminal ileum, protruding deeply in the pelvic floor, were identified. The ECF was identified in the terminal ileum medially to the loop ileostomy. The cecum, the proximal ascending colon and approximately 1 m of terminal ileum, including the loop ileostomy, was resected; an ileo‐ascending colon anastomosis with a circular stapler (d:28 mm) and a new loop ileostomy was constructed. Subsequently, a biological mesh (non‐cross‐linked porcine dermal collagen, thickness: 2 mm) was secured with absorbable stitches Vicryl 3‐0 to the pelvic outlet (Fig. 2). The postoperative period was uncomplicated, and 8 days later, the patient was discharged. At 4 months follow‐up, the patient remains well without evidence of recurrence.

## Discussion

Anastomotic recurrences following colorectal surgery or anastomotic leaks are common causes of multiple operations in a single patient. Multiple abdominal operations confer to increased morbidity rates; incisional hernias, adhesion‐related bowel obstructions, abdominal collections, and fistulas 5, 6, 7. Adjuvant radiotherapy and multiple episodes of fecal peritonitis following anastomotic leaks and recurrences create a very hostile environment both for the surgeons and for grafts. Biological meshes have proved to be effective in contaminated fields even in cases of abdominal infections with multiresistant microorganisms 4, 8. However, the evidence regarding the use of biological meshes in patients following surgery for colorectal cancer and radiotherapy or the development of ECFs is scarce.

The use of biological meshes on fistulas has been discussed mostly in colorectal and ENT operations; the recurrence rates for oronasal fistulas (ONF) following cleft palate (CP) repair had been high prior to the advent of biological prostheses. However, the acellular dermal matrix allografts have proved to be safe and effective 9. Regarding colorectal surgery, the closure of abdominal wall defects or pelvic outlet following the repair of ECF and complex colorectal operations is still a challenge even for experienced surgeons. In our case, the radical debridement of the pelvis resulted to the protrusion of small bowel loops through the pelvic outlet and the creation of an ECF at the perineum. The reduction in the protruded loops to the abdominal cavity and the closure of the pelvic defect with the application of a biological mesh rendered the resection of the perineal skin unnecessary.

The biological meshes have proved efficient in contaminated or inflamed areas following bowel resection for ECF or radiotherapy for recurrent malignancies with or without the utilization of a superior gluteal artery perforator (SGAP) flap 1. These flaps can be used for the fulfillment of the space between the stitched mesh and the perineal skin 1. However, the combination of the mesh and the flap demands a high level of expertise and experience; the operative time ranges from 10 to 12" h in small case series 1, 4. The existing evidence indicates that risks for graft rejection and other mesh‐related complications are rare. Of note, bevacizumab is associated with increased risk of fistula formation in postoperative cancer patients, and refistulaization has only been described after therapy with this chemotherapy regimen 10, 11. In our case, the patient did not receive this specific agent or any antiangiogenic therapy.

Formation of enteroperineal fistulas is most likely to occur following multiple operations in the lower gastrointestinal tract. Application of biological meshes may prove beneficial in these scenarios as they provide a stable ground for closing pelvic defects even in contaminated fields.

## Authorship

MCM: treated the patient and wrote the manuscript. MK: treated the patient and helped to collect the patient documents. AK: treated the patients and made the literature search. DM: reviewed the draft and made critical revisions. DIT: made critical revisions and prepared the figures. EM: treated the patient and collected patient documents. AL: treated the patient and made the literature search. VK: treated the patient and wrote the manuscript. CD: reviewed the draft and made critical revisions. AZ: treated the patient and wrote the manuscript.

## Conflict of Interest

None declared.
